# Residential Traffic-Related Pollution Exposures and Exhaled Nitric Oxide in the Children’s Health Study

**DOI:** 10.1289/ehp.1103516

**Published:** 2011-06-27

**Authors:** Sandrah P. Eckel, Kiros Berhane, Muhammad T. Salam, Edward B. Rappaport, William S. Linn, Theresa M. Bastain, Yue Zhang, Frederick Lurmann, Edward L. Avol, Frank D. Gilliland

**Affiliations:** 1 University of Southern California, Los Angeles, California, USA; 2 Sonoma Technology Inc., Petaluma, California, USA

**Keywords:** air pollution, airway inflammation, children’s respiratory health, exhaled nitric oxide, traffic

## Abstract

Background: The fractional concentration of nitric oxide in exhaled air (Fe_NO_) potentially detects airway inflammation related to air pollution exposure. Existing studies have not yet provided conclusive evidence on the association of Fe_NO_ with traffic-related pollution (TRP).

Objectives: We evaluated the association of Fe_NO_ with residential TRP exposure in a large cohort of children.

Methods: We related Fe_NO_ measured on 2,143 children (ages 7–11 years) who participated in the Southern California Children’s Health Study (CHS) to five classes of metrics of residential TRP: distances to freeways and major roads; length of all and local roads within circular buffers around the home; traffic densities within buffers; annual average line source dispersion modeled nitrogen oxides (NO_x_) from freeways and nonfreeway roads; and predicted annual average nitrogen oxide, nitrogen dioxide, and NO_x_ from a model based on intracommunity sampling in the CHS.

Results: In children with asthma, length of roads was positively associated with Fe_NO_, with stronger associations in smaller buffers [46.7%; 95% confidence interval (CI), 14.3–88.4], 12.4% (95% CI, –8.8 to 38.4), and 4.1% (95% CI, –14.6 to 26.8) higher Fe_NO_ for 100-, 300-, and 1,000-m increases in the length of all roads in 50-, 100-, and 200-m buffers, respectively. Other TRP metrics were not significantly associated with Fe_NO_, even though the study design was powered to detect exposures explaining as little as 0.4% of the variation in natural log-transformed Fe_NO_ (*R*^2^ = 0.004).

Conclusion: Length of road was the only indicator of residential TRP exposure associated with airway inflammation in children with asthma, as measured by Fe_NO_.

The effects of air pollution on children’s respiratory health (Brunekreef and Holgate 2002) are important because reduced lung function growth and asthma early in life may have lifelong effects (Gauderman et al. 2000). One pathophysiologic mechanism by which air pollution is thought to affect respiratory health is airway inflammation. The fractional concentration of nitric oxide in exhaled air (Fe_NO_) is a noninvasive marker of aspects of airway inflammation (Baraldi and de Jongste 2002; Kharitonov and Barnes 2002; Piacentini et al. 1999) that has been associated with air pollution exposure (Fischer et al. 2002; Koenig et al. 2003). Several studies have examined the association of traffic-related pollutants (TRPs) with Fe_NO_ in children (Delfino et al. 2006; Holguin et al. 2007; Steerenberg et al. 2001), but results have not been conclusive. Many studies use different TRP metrics, and only one involves a large number of children (Dales et al. 2008).

The Southern California Children’s Health Study (CHS) is an ongoing prospective cohort study designed to study the chronic effects of air pollution on children’s respiratory health. Traffic plays an important role in Southern California air pollution. TRP has been associated previously with respiratory health in the CHS. Residential proximity to freeways was associated with substantial deficits in lung function growth, independent of regional pollutant effects (Gauderman et al. 2007); residential proximity to major roads and line-source dispersion modeled pollutants were associated with increased risk of asthma and wheeze (McConnell et al. 2006); annual average line-source dispersion modeled pollutants at homes and at schools were associated with increased risk of asthma (McConnell et al. 2010a); and longer school commute time (as a marker for on-road exposure) was associated with increased odds of severe wheezing among children with asthma (McConnell et al. 2010b). In the CHS, short-term increases in community-level ambient particulate matter (PM) ≤ 2.5 and ≤ 10 µm in aerodynamic diameter (PM_2.5_ and PM_10_, respectively), and ozone (O_3_) were associated with elevated Fe_NO_ (Berhane et al. 2011), and elevated Fe_NO_ has been associated with increased risk for incident asthma (Bastain et al. 2010).

The objective of this study was to evaluate the association of Fe_NO_ with five classes of metrics of residential TRP exposure in a large cohort of children.

## Methods

*Study population.* Study participants were children from a CHS cohort enrolled from kindergarten or first-grade classrooms in 2002–2003 in 12 communities in Southern California (a 13th community, Lake Gregory, was excluded because of lack of information on TRP), using a protocol approved by the University of Southern California Institutional Review Board. Informed consent was obtained from a parent or guardian, who completed baseline and annual follow-up written questionnaires, and informed assent was obtained from each child. More information on the study design is available elsewhere (McConnell et al. 2006).

*Fe_NO_ assessment.* Detailed descriptions of Fe_NO_ collection in the CHS have been reported previously (Bastain et al. 2010; Linn et al. 2009a, 2009b). Briefly, Fe_NO_ was collected at schools from October to June during the 2005–2006 school year using an offline breath collection technique according to American Thoracic Society (ATS) guidelines [ATS 1999; ATS/European Respiratory Society (ATS/ERS) 2005]. Collection occurred primarily in the mid to late morning to minimize possible effects of early morning traffic-related peaks in ambient nitrogen oxide (NO) and recent food intake. Participants with acute respiratory infection in the preceding 3 days were rescheduled or excluded. To differentiate seasonal and spatial effects, each CHS community was visited at least twice in different seasons. In subsequent study years, online Fe_NO_ collection, which allows real-time flow monitoring and is not subject to NO measurement errors related to analysis delay or bagged sample contamination, became feasible in a large study population. A pilot study with collection of offline (100 mL/sec flow) Fe_NO_ and measurement of online (50 mL/sec flow, collected according to ATS/ERS guidelines) Fe_NO_ within 4 hr of each other in 2006–2007 was used to develop a model that reliably predicted online values of Fe_NO_ (*R*^2^ = 0.94) using measured offline Fe_NO_, concurrent ambient NO, and offline sample analysis interval (Linn et al. 2009a). In this study, we used the predicted values for online Fe_NO_ at 50 mL/sec flow—similar to previous CHS studies (Bastain et al. 2010; Berhane et al. 2011)—from 2005 to 2006. This year had information on Fe_NO_ for the largest number of children, and Fe_NO_ collection occurred in conjunction with a large exposure measurement campaign designed to quantify intracommunity variation of local ambient pollutants (Franklin et al., in press).

*Residential TRP exposures.* We characterized exposure to residential TRP using five classes of metrics. Distances in meters to the nearest freeway and to the nearest nonfreeway major road were obtained by geocoding residential addresses, as described previously (McConnell et al. 2006). The total length of roads (meters) within circular buffers with radii of 50 m, 100 m, and 200 m centered at the participants’ residences were calculated using TeleAtlas MultiNet road class data (TeleAtlas 2002). Local roads lengths were obtained using data only from the roads classified as major or minor collectors corresponding to functional road class (FRC) 5 or FRC6, respectively. Traffic density (distance-decayed vehicles per day) with 150-m and 300-m falloff radii of the participants’ residences were calculated using additional information on average annual daily traffic assigned to TeleAtlas MultiNet roadway links as described previously (Gauderman et al. 2005). Predicted annual average nitrogen oxides (NO_x_) (in parts per billion) from freeway and nonfreeway roads at participant residence locations were obtained via the California Line-Source Dispersion Model (CALINE4) using information on roadway geometry, traffic volumes, wind speed and direction, atmospheric stability, mixing heights and vehicle emission rates, as described elsewhere (Benson 1989; McConnell et al. 2006). The Intra-Community Variability (ICV) study sampled NO and nitrogen dioxide (NO_2_) at 942 CHS participant resident locations, schools, and central sites across the same 12 CHS communities considered here for 2 weeks in the summer and 2 weeks in the winter of 2005 (Franklin et al., in press). As part of the ICV study, these measurements were used to develop a prediction model for annual average NO (adjusted *R*^2^ = 0.75), NO_2_ (adjusted *R*^2^ = 0.67), and NO_x_ (adjusted *R*^2^ = 0.75) at CHS participants’ homes, with the following information as model inputs: CALINE4 NO_x_ estimates from freeways and nonfreeways, distances to freeways and nonfreeway major roads, population density, elevation, and whether the community was inside the Los Angeles basin.

*Covariate information.* Parent/guardian responses to a written questionnaire during the 2005–2006 school year provided information on race/ethnicity, highest attained parental education, physician diagnosis of asthma, rhinitis, asthma medication use in the previous 12 months, and exposure to secondhand smoke. Height and weight measured on the day of the Fe_NO_ test were used to calculate age- and sex-specific body mass index (BMI) percentiles from Centers for Disease Control and Prevention (CDC) growth charts (CDC 2009).

*Exclusion criteria.* In the 2005–2006 school year, Fe_NO_ was measured on 2,709 participants who provided questionnaire data. We excluded 52 participants whose addresses could not be geocoded with the highest-quality match code and 331participants without information on all TRP exposure metrics. Because inhaled corticosteroid (ICS) medication is known to acutely affect Fe_NO_ levels (Beck-Ripp et al. 2002), we additionally excluded 90 participants who reported taking ICS medication within the previous 12 months and 93 participants who provided no information on medication use. The final analysis data set included 2,143 participants.

*Statistical analysis.* We performed exploratory and descriptive data analyses to summarize the characteristics of the study population and the distributions of the TRP exposure metrics (henceforth referred to as exposures). We calculated within-community correlations of the exposures by subtracting community-specific means from each exposure and then calculating the Pearson’s correlation of the resultant deviations from community-specific means. We used multiple linear regression models to relate natural log (ln)-transformed Fe_NO_ to exposures because Fe_NO_ has a right-skewed distribution. After careful consideration of potential confounders and effect modifiers, all models were adjusted for child’s race/ethnicity, sex, asthma status, use of asthma medication (controller and/or rescue) in the previous 12 months, rhinitis history (never, not current, or current), age at Fe_NO_ collection, BMI percentile, secondhand tobacco smoke, parental education, month and hour of Fe_NO_ collection, whether the Fe_NO_ test was performed outdoors, and community of residence (to control for factors that vary by community, such as regional air pollution). We investigated potential effect modification by asthma status by fitting models with an appropriate interaction term and by fitting separate models for children with and without asthma. Because many of the exposures were correlated, we fit single-pollutant models.

To investigate possible nonlinear exposure–response relationships, we fit generalized additive models (Hastie and Tibshirani 1990) to assess the functional relationship of each exposure metric with ln(Fe_NO_), using a procedure that estimates the degrees of freedom of the smooth relationship as part of the model-fitting process (Wood 2006). The adjustment variables were the same as in the linear regression.

We applied an indicator variable approach to address the small proportion of missing data on the adjustment covariates (5.6% of study participants were missing data on at least one covariate). Our results were not sensitive to this approach, because complete case or multiple imputation analyses produced similar results (data not shown).

We performed additional sensitivity analyses by *a*) testing for heterogeneity in TRP effects by race/ethnicity; *b*) additionally adjusting for recent pollution: ambient NO at the time of test at the testing location, daily community-specific central site 24-hr cumulative lagged average of PM_2.5_ (over 1–8 days) (Berhane et al. 2011), or central site ambient O_3_, NO_2_, or PM_2.5_ on the day of or 1 or 2 days before the test or the average of the 2 days before the test; and *c*) restricting the analysis to the subset of children reporting no change of residence since November 2004.

To determine the lower bound of effects detectable at 80% power with our study design, we performed a power simulation study [see Supplemental Material, Power Simulation (http://dx.doi.org/10.1289/ehp.1103516)] using information from this study on sample size, the distribution of Fe_NO_ and its association with adjustment covariates, and the distributions of the TRP variables.

Analyses and simulations were performed using R statistical software (R Project for Statistical Computing, Vienna, Austria). All hypothesis tests used a two-sided alternative and a 0.05 significance level.

## Results

*Participant characteristics.* The children were between 7 and 11 years old; slightly more than half were female, a majority reported an ethnicity of Hispanic, and 5.0% were exposed to secondhand smoke. Fe_NO_ was right-skewed and ranged from 2.8 to 176.3 ppb, with a geometric mean and standard deviation of 13.3 and 1.9 ppb, respectively. Compared with children without asthma, those with asthma had higher Fe_NO_, more often were male, and more often had current respiratory allergy (Table 1). Fe_NO_ levels varied significantly between CHS communities (*p* < 0.001), with the highest geometric mean observed in Long Beach (16.4 ppb) and the lowest in Glendora (10.6 ppb).

**Table 1:** Demographic characteristics and potentialconfounders by parent report of doctor-diagnosed asthma.

Without asthma (*n* = 1,934)	With asthma (*n* = 209)
Characteristic	Mean ± SD or %	Mean ± SD or %
Fe_NO_ (ppb)*a*		13.1 (1.8)		16.2 (2.1)
Age (years)		9.3 ± 0.6		9.3 ± 0.6
Percent male		47.1		55.5
Body mass index percentile		66.0 ± 29.2		69.7 ± 28.7
Percent missing		0.6		0.5
Race/ethnicity (%)				
White/non-Hispanic		33.7		32.1
Hispanic		56.8		55.0
Black		1.8		2.9
Asian/Hawaiian/Pacific Islander		3.1		3.3
Other		4.6		6.7
Missing		0.1		0.0
Parent education (%)				
< 12th grade		20.4		15.3
Completed 12th grade		17.1		16.7
> 12th grade		58.0		64.6
Missing		4.5		3.3
Respiratory allergy (%)				
Never		47.6		17.7
No current		28.3		29.7
Current		24.0		52.6
Missing		0.1		0.0
Asthma medication (%)				
None		97.4		62.7
Rescue only		2.2		28.2
Control only		0.2		3.3
Rescue and control		0.2		5.7
Exposed to secondhand smoke (%)		5.1		3.8
Missing		0.6		1.4
Time of Fe_NO_ collection [hours (%)]				
0800–0859		6.3		5.3
0900–1159		85.7		81.8
1200–1359		7.4		12.0
1400–1559		0.7		1.0
Percent outdoor test		3.2		2.4
**a**Geometric mean (SD).				

*TRP distributions and correlations.* Most TRP metrics were right-skewed, except for length of road and the ICV prediction of annual average NO_2_, which were more symmetrically distributed (Table 2). Approximately 22% of the children had 50-m buffer length of road values of 99–100 m because of the unique geometry of having a single road set back 10 m from the residence location at the center of the small buffer. On average, local roads contributed most to the total length of roads, particularly in smaller buffers [see Supplemental Material, Figure 1 (http://dx.doi.org/10.1289/ehp.1103516)]. Within communities, distances to freeway and major road were moderately correlated (0.42), traffic densities within 150-m and 300-m buffers were highly correlated (0.90), the lengths of all and local roads had correlations of 0.61 to 0.72, CALINE4 predictions of freeway and nonfreeway NO_x_ had low correlation (0.05), and ICV predictions of NO, NO_2_, and NO_x_ were highly correlated (0.91 to 0.98) (Table 3). Length of road generally had low correlation (< 0.38) with the other TRP metrics.

**Table 2:** Distribution summaries for the TRP exposure metrics.

Percentile								
Exposure	Mean ± SD		Min	5th	25th	50th	75th	95th	Max
Distance: freeway (m)		1469.7 ±	1200.5		23.8		131.6		483.9		1167.2		2191.4		3723.0		8567.4
Distance: major road (m)		462.1 ±	533.1		3.0		7.8		140.0		294.1		585.9		1541.0		5642.3
Length all roads: 50-m buffer (m)		126.8 ±	48.7		0.0		75.3		99.3		106.5		153.6		204.8		408.0
Length local roads: 50-m buffer (m)		114.4 ±	47.9		0.0		0.0		98.2		99.9		145.1		190.9		302.4
Length all roads: 100-m buffer (m)		406.4 ±	162.0		0.0		196.8		299.6		393.5		507.3		688.0		1072.0
Length local roads: 100-m buffer (m)		356.8 ±	153.3		0.0		120.4		247.7		356.2		460.9		607.9		971.7
Length all roads: 200-m buffer (m)		1552.0 ±	565.7		0.0		572.7		1175.2		1582.2		1928.0		2454.1		3311.0
Length local roads: 200-m buffer (m)		1313.9 ±	511.8		0.0		421.5		971.5		1356.5		1682.4		2086.0		3090.7
Density: 150-m buffer (vehicles/day)		9199.1 ±	19562.2		0.0		0.0		0.0		2285.5		10429.3		34902.3		195515.8
Density: 300-m buffer (vehicles/day)		16435.5 ±	28963.6		0.0		0.0		870.7		6348.1		18716.4		71089.3		224321.4
CALINE4 NO_x_: freeway (ppb)		13.3 ±	15.4		0.0		1.1		3.3		9.0		17.8		38.9		197.0
CALINE4 NO_x_: nonfreeway (ppb)		6.7 ±	5.9		0.0		0.9		2.8		5.2		9.3		16.9		49.8
Predicted NO (intracommunity)		17.9 ±	14.4		0.4		2.7		7.2		13.3		23.8		47.9		78.3
Predicted NO_2_ (intracommunity)		19.4 ±	8.8		2.9		4.9		11.1		20.7		26.7		31.6		41.4
Predicted NO_x_ (intracommunity)		37.4 ±	21.8		4.0		8.4		19.2		34.0		51.6		77.3		109.0
Abbreviations: Max, maximum; Min, minimum.																	

**Figure 1:**
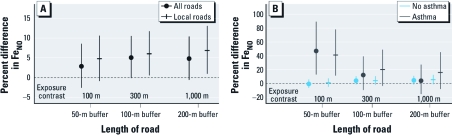
Estimated percent difference in Fe_NO_*^a^* and 95% CI associated with an increase*^b^* in each length of road metric: (*A*) adjusting for asthma status and (*B*) fitting separate models by asthma status. ***^a^***Adjusted for race/ethnicity, sex, asthma status (*A*), asthma medication, rhinitis history, age at collection, BMI percentile, secondhand tobacco smoke, parental education, month and hour of Fe_NO_ collection, outdoor testing, and community of residence. ***^b^***Exposure contrasts: 100 m, 300 m, and 1,000 m for length of roads in a 50-m, 100-m, and 200-m buffer, respectively.

**Table 3:** Within-community correlations of the TRP exposure metrics.

Distance			Length: 50 m			Length: 100 m			Length: 200 m			Density			CALINE4			Predicted				
Exposure	Fwy	M road	All	Local	All	Local	All	Local	150 m	300 m	Fwy	Nonfwy	NO	NO_2_	NO_x_
Distance: freeway		1.00																												
Distance: major road		0.42		1.00																										
Length all roads: 50-m buffer		–0.11		–0.14		1.00																								
Length local roads: 50-m buffer		–0.04		0.07		0.61		1.00																						
Length all roads: 100-m buffer		–0.17		–0.20		0.62		0.40		1.00																				
Length local roads: 100-m buffer		–0.06		0.03		0.38		0.69		0.72		1.00																		
Length all roads: 200-m buffer		–0.26		–0.30		0.40		0.29		0.73		0.55		1.00																
Length local roads: 200-m buffer		–0.08		–0.01		0.28		0.49		0.54		0.77		0.72		1.00														
Density: 150-m buffer		–0.22		–0.14		0.15		–0.07		0.29		–0.08		0.31		–0.09		1.00												
Density: 300-m buffer		–0.30		–0.16		0.11		–0.02		0.23		–0.05		0.35		–0.09		0.90		1.00										
CALINE4 NO_x_: freeway		–0.48		–0.15		0.04		0.03		0.13		0.01		0.22		–0.01		0.59		0.70		1.00								
CALINE4 NO_x_: nonfreeway		–0.10		–0.25		0.38		–0.17		0.25		–0.15		0.22		–0.07		0.26		0.18		0.05		1.00						
Predicted NO		–0.44		–0.31		0.29		–0.10		0.26		–0.08		0.32		–0.04		0.49		0.51		0.57		0.69		1.00				
Predicted NO_2_		–0.50		–0.41		0.26		–0.05		0.25		–0.03		0.34		0.03		0.38		0.42		0.54		0.67		0.91		1.00		
Predicted NO_x_		–0.49		–0.37		0.27		–0.07		0.25		–0.05		0.34		–0.01		0.45		0.49		0.59		0.69		0.97		0.98		1.00


*TRP–Fe_NO_ associations.* Length of road was the only class of TRP exposure metric that had any statistically significant associations with Fe_NO_ in all children (Table 4), with slightly stronger positive associations for local roads only compared with all roads (Figure 1A). A large proportion of the variability in Fe_NO_ remained unexplained by our models. A model fit on data from all children with only the adjustment covariates had an *R*^2^ of 0.127 (or 0.233 for a model fit only on children with asthma). The maximum *R*^2^ of a model that included an additional linear effect of a single TRP metric was 0.130 (0.273 for children with asthma). For children with asthma, there was a positive association of length of road with Fe_NO_, with a stronger and statistically significant association in the 50-m buffer (Figure 1B). For children without asthma, there was no statistically significant association of any length of road metric with Fe_NO_. Specifically, the estimated percent difference in Fe_NO_ associated with a 100-m, 300-m, and 1,000-m increase in the length of all roads in a 50-m, 100-m, and 200-m buffer was 46.7 [95% confidence interval (CI), 14.3 to 88.4], 12.4 (95% CI, –8.8 to 38.4), and 4.1 (95% CI, –14.6 to 26.8), respectively, for children with asthma and –0.2 (95% CI, –5.5 to 5.3), 4.6 (95% CI, –0.6 to 10.0), and 4.7 (95% CI, –0.8 to 10.4), respectively, for children without asthma.

**Table 4:** Estimated percent difference in Fe_NO_*a* associated with an increase*b* in each TRP exposure metric.

Exposure	Percent difference (95% CI)		*p*-Value
Distance: freeway		–0.15	(–1.48 to 1.21)		0.83
Distance: major road		–0.72	(–1.82 to 0.39)		0.20
Length all roads: 50-m buffer		2.84	(–2.45 to 8.42)		0.30
Length local roads: 50-m buffer		4.75	(–0.68 to 10.49)		0.09
Length all roads: 100-m buffer		5.07	(0.03 to 10.37)		0.05
Length local roads: 100-m buffer		6.02	(0.72 to 11.60)		0.03
Length all roads: 200-m buffer		4.80	(–0.42 to 10.29)		0.07
Length local roads: 200-m buffer		6.84	(1.10 to 12.90)		0.02
Density: 150-m buffer		0.36	(–1.00 to 1.73)		0.61
Density: 300-m buffer		0.06	(–0.88 to 1.01)		0.90
CALINE4 NO_x_: freeway		–0.32	(–1.38 to 0.76)		0.56
CALINE4 NO_x_: nonfreeway		–0.63	(–3.39 to 2.21)		0.66
Predicted NO		–2.59	(–6.88 to 1.90)		0.25
Predicted NO_2_		–1.17	(–8.60 to 6.87)		0.77
Predicted NO_x_		–1.08	(–3.98 to 1.90)		0.48
**a**Adjusted for race/ethnicity, sex, asthma status, asthma medication, rhinitis history, age at collection, BMI percentile, secondhand tobacco smoke, parental education, month and hour of Fe_NO_ collection, outdoor testing, and community of residence. **b**Exposure contrasts: 500 m for distance to freeway; 200 m for distance to major road; 100 m, 300 m, and 1,000 m for length of roads in a 50-m, 100-m, and 200-m buffer, respectively; 10,000 vehicles/day for traffic densities; 5 ppb for CALINE4 predicted NO_x_; and 10 ppb for intracommunity predictions of NO, NO_2_, and NO_x_.					

**Figure 2:**
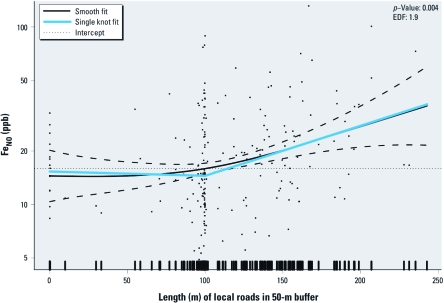
Smooth association of length of local roads in a 50-m buffer with Fe_NO_ for children with asthma*^a^* (black solid line: smooth fitted value for a child with average values for the adjustment covariates; black dashed line is the 95% CI). The estimated degrees of freedom (EDF) and the *p*-value testing the association of the smooth with Fe_NO_ are displayed. On the *y*-axis, values are plotted on the natural log scale and labeled on the original scale. ***^a^***Adjusted for race/ethnicity, sex, asthma medication, rhinitis history, age at collection, BMI percentile, secondhand tobacco smoke, parental education, month and hour of Fe_NO_ collection, outdoor testing, and community of residence.

*Nonlinear TRP–Fe_NO_ associations.* For children with asthma, Fe_NO_ had a nonlinear association with the length of local roads in a 50-m buffer. There was no evidence for an association of length of road with Fe_NO_ for shorter lengths of road, but a strong positive association when the length of road was longer than 100 m (Figure 2). Using a linear spline with a single knot at 100 m to approximate the smooth function, we estimated that for children with asthma who have > 100 m of local roadways in a 50-m buffer, a 50-m increase in local road length is associated with a 38.3% increase in Fe_NO_ (95% CI, 16.1 to 64.8), more than twice the estimated effect for a 50-m increase when we assume the relationship is linear over the range of the data (18.7; 95% CI, 5.9 to 33.1). There was no evidence of other biologically relevant nonlinear exposure–response relationships between the other TRP metrics and Fe_NO_ (data not shown).

*Sensitivity analyses.* There was no evidence of heterogeneity in TRP effects by race/ethnicity or community. The associations of length of road with Fe_NO_ were generally stronger in the subgroup of 1,937 children who had not reported a move since November 2004 (data not shown) and not sensitive to adjustment for recent pollution, including ambient NO at the time of the test.

*Power simulation study.* A study with our design has 80% power to detect an exposure that explains as little as 0.4% of the variation in ln(Fe_NO_) (*R*^2^ = 0.004) after controlling for the adjustment covariates. The minimum detectable effect sizes for the given exposure contrasts ranged from a percent difference in Fe_NO_ of 1.15 for a 5-ppb increase in CALINE4 estimated annual average freeway NO_x_ to 7.66 for a 100-m increase in the length of local roads in a 50-m buffer [see Supplemental Material, Table 1 (http://dx.doi.org/10.1289/ehp.1103516)]. As expected (Piantadosi 2005), the estimated TRP effects in this study are generally smaller than the minimum detectable effect sizes, except for the length of road metrics.

## Discussion

Length of road was the only residential TRP metric associated with Fe_NO_. The strongest significant associations were observed in small buffers for children with asthma, replicating findings in other studies. Sensitivity analyses restricting the analysis to children who had not moved in the previous year or additionally adjusting for short-term ambient pollution yielded similar results, confirming the findings. To the best of our knowledge, this study of many indicators of TRP and Fe_NO_ in children is the largest to date.

The largest comparable study related Fe_NO_ in 1,613 children in the single community of Windsor, Ontario, Canada to *a*) land-use regression–modeled annual averages of NO_2_, SO_2_, coarse PM, PM_2.5_, and black smoke at the residential postal code; *b*) distance to a single major truck transportation route; and *c*) length of all or local roadways within 200 m of the residence (Dales et al. 2008). Fe_NO_ was associated with length of all (*p* < 0.01) and local (*p* < 0.05) roadways. In our study, we had no metric analogous to distance to truck route, but Fe_NO_ was highest in Long Beach, California, a community with more truck traffic. We had finer spatial resolution and a wider range for annual average NO_2_ but comparable distributions of lengths of roads in a 200-m buffer. The estimated percent difference in Fe_NO_ associated with a 1,000-m increase in the length of local roads in a 200-m buffer in our study (6.8; 95% CI, 1.1 to 12.9) was similar to the estimate in Windsor (6.8; 95% CI, 0.2 to 13.9). In Windsor, the length of all roads in a 200-m buffer was statistically significantly associated with Fe_NO_. We observed smaller, nonsignificant associations [see Supplemental Material, Figure 2 (http://dx.doi.org/10.1289/ehp.1103516)].

A study of 200 children in Mexico, half with asthma, examined the association of Fe_NO_ with length of road and traffic densities within 50-m, 100-m, 200-m, 300-m, 400-m, 500-m, and 750-m buffers around schools and homes (Holguin et al. 2007). Statistically significant associations of Fe_NO_ with residential length of road were found only for children with asthma. The associations were strongest in the smallest buffers. Results from our study were qualitatively similar, with tighter CIs as expected from our larger sample size [see Supplemental Material, Figure 2 (http://dx.doi.org/10.1289/ehp.1103516)]. For example, in a residential 50-m buffer, the percent difference in Fe_NO_ associated with a 100-m increase in the length of all roads for children with asthma was 46.7 (95% CI, 14.3 to 88.4) in our study and 47.9 (95% CI, 5.0 to 108.2) in Mexico.

A study of 82 children found offline Fe_NO_ to be 8.8% higher (95% CI, –7 to 58) in urban children compared with suburban children, a difference possibly related to TRP exposure but potentially confounded by the lack of adjustment for ambient NO at the time of the test (Steerenberg et al. 2001). A study of 812 Dutch schoolchildren found offline Fe_NO_ to be statistically significantly associated with recent PM_10_ (0–3 days before the test) but not with distance from a motorway or with traffic counts, although a larger positive association (not significant) was observed for children with asthma (Graveland et al. 2010). Two smaller studies of children found short-term increases in ambient NO_x_ (*n* = 19) (Murata et al. 2007) and personal NO_2_ (*n* = 45) (Delfino et al. 2006) to be associated with elevated Fe_NO_, whereas a third study found no association with short-term ambient NO_2_ in 182 children with asthma (Liu et al. 2009). Fourteen nonsmoking adults with mild asthma had no significant differences in Fe_NO_ after exposure to rush-hour traffic in a tunnel (Larsson et al. 2010).

This study has several strengths. It is a large, ongoing, prospective cohort study that included ethnically diverse children—with and without asthma—in 12 communities in Southern California, an area with a uniquely broad range of air pollution exposures in which TRP plays an important role. Multiple metrics were available to measure different features of TRP exposure. Distance, total length of road, and traffic density offered straightforward, although somewhat crude, measures of the effects of proximity to roadways and may be indicators of short- or long-term exposure. CALINE4 predictions accounted for key factors that determine exposure, such as wind speed and direction, and the CHS ICV study predictions offered a further refinement of the exposure surface. However, both predictions focused only on annual averages of specific TRP components that have been considered as representative surrogates for products of traffic-related combustion (Brunekreef and Holgate 2002) and did not, for example, model ultrafine particles.

This study also has several limitations. We were unable to disentangle the effects of asthma medication use on the TRP–Fe_NO_ association. We had information only on parent report of asthma medication use in the previous 12 months, so we excluded participants taking ICS medication. Information on recent food intake or exercise was not available, but we adjusted for time of day of collection. We adjusted for parent education, but because socioeconomic status (SES) may be related to TRP exposure, there is a potential for residual confounding by SES. However, results were similar when we additionally adjusted for household income and whether the child had health insurance. We conducted thorough exploration during model building and sensitivity analyses, but as in any analysis of observational data, we may have lacked data on or been unaware of other potentially important confounding variables. Asthma is an important susceptibility factor; our determination of asthma status by parent report of doctor diagnosis has limitations but is widely used in epidemiologic studies (Burr 1992). We had limited data on time–activity patterns. The potential for resultant exposure misclassification may be reduced by the long-term characterization of many of the TRP metrics. Future work improving exposure assignment would be beneficial [see Supplemental Material for a discussion on length of road (http://dx.doi.org/10.1289/ehp.1103516)].

Length of road was the only TRP metric associated with Fe_NO_ in our study population. For other metrics, we can compare minimum detectable effect sizes based on our study design with effect sizes observed in other studies. For example, Dales et al. (2008) estimated a 4.0% (95% CI, –10.2 to 20.6) difference in Fe_NO_ associated with a 10-ppb increase in land-use regression–modeled annual average NO_2_. Our study design had 80% power to detect an association of similar magnitude (a 4.1% difference in Fe_NO_ per 10-ppb increase in ICV predicted annual average NO_2_), assuming similarity in these metrics across studies. However, in our study—with larger sample size and greater exposure contrast—we observed an effect that was smaller and negative (–1.2; 95% CI, –8.6 to 6.9). This result, along with our other findings, is consistent with the null hypotheses that local, long-term average NO_2_, NO, NO_x_ exposures; local traffic densities; and distance to freeway and major road are not associated with Fe_NO_ in our study population. Similarly, in a previous study, we found evidence of short-term but not long-term effects of community-level ambient PM_2.5_ on Fe_NO_ (Berhane et al. 2011).

Length of road had little correlation with the other TRP metrics, potentially signifying that it captures information on a different, relevant feature of TRP. It would be useful scientifically and from a public health perspective to identify this feature. Length of road predicts soil lead levels in Los Angeles (Wu et al. 2010) and ambient pollution in land-use regression models (Hoek et al. 2008) and is associated with acute respiratory illness requiring a hospital visit in children with asthma (Chang et al. 2009). Length of road may better represent exposure to TRP than traffic density or dispersion models because of limited information on traffic counts on smaller local roads (Dales et al. 2008; Holguin et al. 2007). In addition, a dense network of local roads may imply proximity to intersections with potentially sharp exposure gradients of combustion products associated with acceleration and brake wear emissions associated with stopping.

## Conclusion

Length of road was the only indicator of residential TRP exposure associated with airway inflammation in children with asthma, as measured by Fe_NO_. This finding is robust and replicates previous studies, warranting further investigation to identify the attributes of on-road activity driving this association.

## Supplemental Material

(216 KB) PDF
