# Supplementary material for: Residential Traffic-Related Pollution Exposures and Exhaled Nitric Oxide in the Children’s Health Study
Source: Environ Health Perspect. 2011 Jun 27;119(10):1472–7. doi: 10.1289/ehp.1103516 (PMC3230449; doi:10.1289/ehp.1103516)
Supplement: (216 KB) PDF [file ehp.1103516.s001.pdf]

Supplemental Material

Residential Traffic-Related Pollution Exposures and Exhaled Nitric Oxide in the Children's Health Study

Sandrah P. Eckel[1], Kiros Berhane[1], Muhammad T. Salam[1], Edward B. Rappaport[1], William S. Linn[1], Theresa M. Bastain[1], Yue Zhang[1], Frederick Lurmann[2], Edward L. Avol[1], Frank D. Gilliland[1]

[1]University of Southern California, Los Angeles, CA, USA;

[2] Sonoma Technology Inc., Petaluma, CA, USA

Correspondence to: Sandrah P. Eckel, PhD, 1540 Alcazar Street, CHP-220, Department of Preventive Medicine, Keck School of Medicine, University of Southern California

Los Angeles, CA 90089-9011, E-mail: eckel@usc.edu; Tel.: 323-442-2030; Fax: 323-442-2349

Table of Contents **Page**

Table of Contents	Page
Supplemental Methods, Power Simulation	3
Supplemental Discussion, Length of Road Metric	4
Supplemental Material, Table 1. Power simulation results	5
Supplemental Material, Figure 1. Proportion of length of all roadways from each roadway type	6
Supplemental Material, Figure 2. Length of road effects compared to those previously reported in other studies	7
References for Supplemental Material	8

Supplemental Methods, Power Simulation

To determine the lower bound of effects detectable at 80% power with our study design, we performed a simulation study. We used information from this study on sample size (N=2,143), the distribution of FeNO (*Y*) and its association with adjustment covariates, as well as the distribution of the TRP (*X*) variables. First, we regressed the natural log (ln) transformed FeNO on the adjustment covariates and saved the estimated variance (σ^2) of the vector of residuals (ε). Separately for each *X*, we then related the residuals to *X* in the linear regression

$$\varepsilon = \beta X + \delta, \qquad (1)$$

where $\delta \sim N(0, \tau^2)$ and β quantifies the association of ln(FeNO) with *X* (an indicator of TRP), after controlling for the adjustment covariates. We selected a set of values of R^2 describing the strength of the association of ln(FeNO) with *X*, after controlling for the adjustment covariates. For each value of R^2, we used the mathematical relationship between R^2, σ^2, and the variance of *X* to determine the corresponding values of β and τ^2. We then generated 5,000 datasets, each containing 2,144 values randomly drawn from the distribution $\varepsilon \sim N(\beta X, \tau^2)$. We fit the linear regression from Equation (1) on each dataset and calculated the power: the proportion of times over the 5,000 datasets that the null hypothesis ($\beta = 0$) was correctly rejected. We then repeated the procedure for the remaining *X*. The minimum detectable effect size was obtained by translating the value of R^2 for which the power was approximately 80% to β.

Supplemental Discussion, Length of Road Metric.

Length of road was calculated using TeleAtlas Multi-net road class data (with improved geographical accuracy compared to the earlier StreetMap product), for which 100% of FRC01 and FRC3 roads are represented as a single direction of travel, FRC4 roads are a mixture of single and bi-directional road links, and approximately 99% of FRC5 and FRC6 roads are bi-directional road links. When calculating the length of all roads in a buffer, larger roads may be included twice (once for each direction of travel), while smaller roads will be included only once. Rather than resulting in erroneous “double counting” the separation of larger roads into two directions of travel may be justified, particularly in small buffers under consideration here (50m, 100m, and 200m), because a typical 5 lane freeway in Los Angeles has a minimum width of approximately 70m. This issue merits further consideration, especially if the length of road metric becomes an increasingly important indicator of traffic-related pollution for which it is of interest to separate the effects of traffic on small local roads from the effects of traffic on larger roads such as freeways.

Supplemental Table

Supplemental Material, Table 1. The absolute value of the minimum detectable percent difference in FeNO[a] associated with an increase[b] in each exposure metric at 80% power, based on 5,000 simulated datasets.

Exposure	Minimum detectable Percent difference
Distance: Freeway	1.48
Distance: Major Road	1.33
Length all roads: 50m buffer	7.52
Length local roads: 50m buffer	7.66
Length all roads: 100m buffer	6.76
Length local roads: 100m buffer	7.16
Length all roads: 200m buffer	6.45
Length local roads: 200m buffer	7.15
Density: 150m buffer	1.82
Density: 300m buffer	1.23
CALINE4 NOx: Freeway	1.15
CALINE4 NOx: Non-Freeway	3.02
Predicted NO	2.49
Predicted NO2	4.11
Predicted NOx	1.64

[a] Adjusted for: race/ethnicity, sex, asthma status, asthma medication, rhinitis history, age at collection, body mass index percentile, second hand tobacco smoke, parental education, month and hour of FeNO collection, outdoor testing, and community of residence.

[b] Exposure contrasts: 500m for distance to freeway; 200m for distance to major road; 100m, 300m, and 1,000m for length of roads in a 50m, 100m, and 200m buffer, respectively; 10,000 vehicles/day for traffic densities; 5 ppb for CALINE4 predicted NO_x; and 10 ppb for intra-community predictions of NO, NO_2, and NO_x.

Supplemental Figures

Supplemental Material, Figure 1. Boxplots[a] of the proportion of length of all roadways (FRC01-6) from each type of roadway (FRC01, FRC3, FRC4, FRC5 and FRC6), by buffer radius. Means are plotted using an "x" symbol.[c]

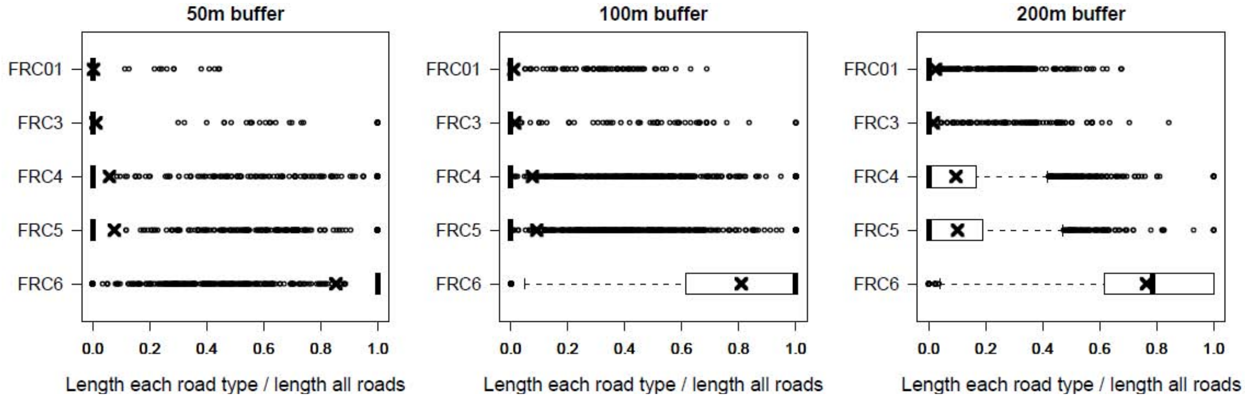

[a] Due to extremely skewed distributions, many of the boxes demarcating the 25th and 75th percentiles appear as degenerate vertical lines.

[b] Functional road classes (FRC) are defined by TeleAtlas Multi-Net as: freeways/highways (FRC01); major arterials (FRC3); minor arterials (FRC4); major collectors (FRC5); and minor collectors (FRC6).

[c] The mean percent of length of road in a 50m buffer from FRC01, FRC3, FRC4, FRC5, and FRC6 roads were: 0.2%, 1.1%, 5.8%, 7.5%, and 85.4%, respectively, while the analogous values for the 200m buffer were: 2.4%, 1.6%, 9.6%, 10.1%, and 76.4%.

Supplemental Material, Figure 2. Estimated percent difference in FeNO[a] and 95% confidence interval associated with an increase[b] in each exposure metric: (a) adjusting for asthma status and (b) fitting separate models by asthma status. Estimates from previous studies are plotted to the right of the corresponding estimates from this study: Dales et al. 2008 in blue with a "D" symbol and Holguin et al. 2007 in green with an "H" symbol.

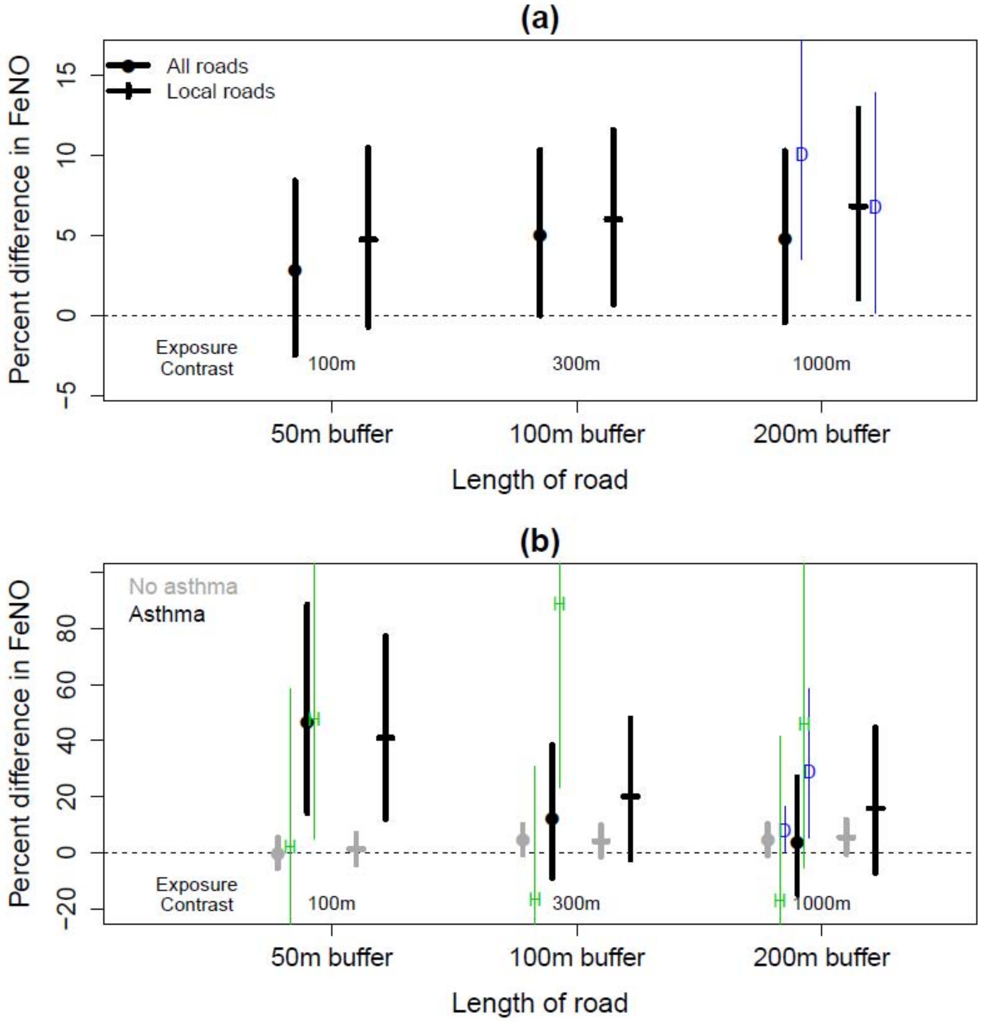

[a] Adjusted for: race/ethnicity, sex, asthma status, asthma medication, rhinitis history, age at collection, body mass index percentile, second hand tobacco smoke, parental education, month and hour of FeNO collection, outdoor testing, and community of residence.

[b] Exposure contrasts: 100m, 300m, and 1,000m for length of roads in a 50m, 100m, and 200m buffer, respectively.

REFERENCES

Dales R, Wheeler A, Mahmud M, Frescura AM, Smith-Doiron M, Nethery E, et al. 2008. The influence of living near roadways on spirometry and exhaled nitric oxide in elementary schoolchildren. Environ Health Perspect 116(10):1423-1427.

Holguin F, Flores S, Ross Z, Cortez M, Molina M, Molina L, et al. 2007. Traffic-related exposures, airway function, inflammation, and respiratory symptoms in children. Am J Respir Crit Care Med 176(12):1236-1242.
